# Boldo leaves reduce seizures, neuroinflammation, and hemichannel activity in a murine model of chronic epilepsy

**DOI:** 10.1186/s40659-025-00647-w

**Published:** 2025-12-07

**Authors:** Claudia García-Rodríguez, Carolina Flores-Muñoz, Paola Fernández, Marcela Escobar, Álvaro O. Ardiles, Ana M. Cardenas, Juan C. Sáez

**Affiliations:** 1 Present Address: Instituto de Neurociencias, Centro Interdisciplinario de Neurociencias de Valparaíso, Universidad de Valparaíso, Valparaíso, Chile; 2 Facultad de Farmacia, Universidad de Valparaíso, Valparaíso, Chile; 3 Facultad de Medicina, Escuela de Medicina, Universidad de Valparaíso, Valparaíso, Chile

**Keywords:** Epilepsy, Neuroinflammation, Boldo, Hemichannel, Pentylenetetrazole, Valproate

## Abstract

Epilepsy is a chronic neurological disorder characterized by a propensity for seizures due to an imbalance between excitatory and inhibitory brain activity. This condition also induces neuroinflammation, which contributes to disease progression. Given that hemichannels (HCs) permeabilize the cell membrane of glia playing a critical role in neuroinflammation, we investigated the antiepileptic potential of Boldo (*Peumus boldus M.*), an endemic Chilean tree containing several bioactive molecules including boldine, a HC inhibitor. Mice were treated with pulverized Boldo leaves, the antiseizure medication valproate, or a combination of both for 5 days. Seizure severity was assessed in a pentylenetetrazole-induced kindling mouse model. Using the dye uptake technique, we evaluated the membrane permeability in hippocampal astrocytes, microglia, and neurons. Additionally, we analyzed astroglial and microglial reactivity and measured levels of pro-inflammatory cytokines (IL-1β, IL6, and TNF-α). Both Boldo and valproate significantly reduced seizure severity. However, distinct mechanisms were observed. Valproate administration increased dye uptake in control animals and enhanced glial reactivity, corroborating its established ability to stimulate hemichannel activity. Conversely, Boldo treatment, either alone or in conjunction with valproate, reduced these parameters, consistent with its HC-blocking properties. Importantly, Boldo was more effective than valproate in reducing plasmatic levels of inflammatory and oxidative stress markers. These findings indicate that Boldo, by inhibiting these HCs, could provide a valuable therapeutic strategy to mitigate neuroinflammation in epilepsy, highlighting the clinical potential of this readily available medicinal herb.

**Graphical abstract:**

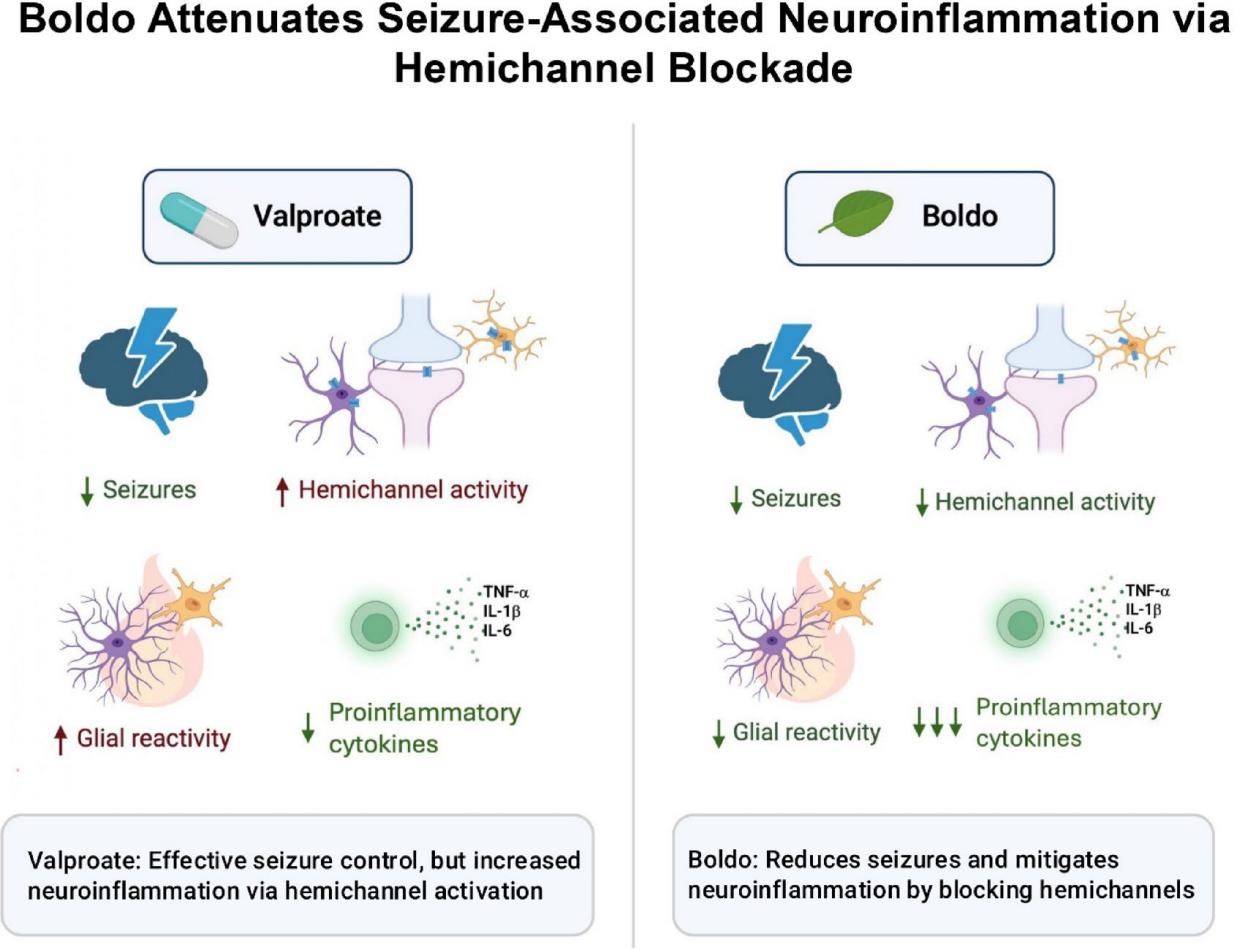

**Supplementary Information:**

The online version contains supplementary material available at 10.1186/s40659-025-00647-w.

## Highlights

- Glial hemichannel activity increases in epilepsy and is worsened by valproate treatment.
- Valproate fails to reduce glial activation and increases the activity of hemichannels.
- Boldo leaves block hemichannels and reduce seizure severity in epileptic mice.
- Boldo lowers proinflammatory markers more effectively than valproate alone.
- Boldo, with its anti-inflammatory properties, offers a novel therapeutic option for epilepsy.

**Supplementary Information:**

The online version contains supplementary material available at 10.1186/s40659-025-00647-w.

## Introduction

Epilepsy, a prevalent neurological disorder, affects an estimated 65–70 million people worldwide, with a notable concentration in low- and middle-income countries [83, 84]. The hallmark of epilepsy is a chronic propensity for seizures, which can manifest in diverse forms, from brief absence seizures to severe tonic–clonic convulsions. These seizures disrupt the delicate balance of excitatory and inhibitory brain activity, leading to aberrant, synchronized electrical discharges and a cascade of neurobiological, cognitive, psychological, and social consequences [28]. This condition is associated with a 2–10% reduction in life expectancy compared to the general population and a fourfold increased risk of comorbidities like depression and anxiety [63]. Etiologies encompass structural, genetic, metabolic, infectious, immune, and idiopathic factors [18]. Epilepsy is also associated with neuroinflammation, a condition contributing to disease progression and potentially drug resistance [76]. This neuroinflammation is driven, in part, by seizures, which stimulate the release of inflammatory cytokines, further promoting neuroinflammation, hyperexcitability, and excitotoxicity [69].

Contemporary antiepileptic therapies predominantly focus on modulating the neuronal excitation/inhibition balance. Specifically, antiseizure medications (ASMs), previously known as antiepileptic drugs, target neuronal ion channels (sodium, potassium, calcium, and chloride channels) to attenuate glutamate-mediated excitatory neurotransmission or augment GABA-mediated inhibitory neurotransmission [9, 56]. This singular focus, however, neglects the potential contributions of other pathophysiological processes, like neuroinflammation. Hemichannels (HCs) and other membrane proteins have been identified as significant players in inflammatory responses and seizure genesis [6, 33]. Furthermore, glial activation is increasingly recognized as a critical component of the tissue response to epileptogenic insults [8, 13, 17]. While ASMs offer relief for many patients, often endure lifelong side effects, and a substantial 30% exhibit drug-resistant epilepsy, necessitating surgical intervention. Consequently, the identification of novel ASMs or the optimization of existing therapeutic strategies is imperative.

Valproate (VPA) is a widely used ASM globally. It is recommended as a first-line monotherapy or adjunctive therapy for generalized tonic–clonic, myoclonic, absence, and unclassified seizures and as a second-line option for focal-onset seizures [41]. It is important to acknowledge that VPA, like other ASMs, can induce a range of systemic and neurological adverse effects, which can significantly impair patients' quality of life [41]. In addition to its use in epilepsy, VPA is also utilized in the management of psychiatric disorders, such as manic states, bipolar, panic, and schizophreniform disorders, and the prevention and acute treatment of migraines [50]. The wide range of applications for VPA can be attributed to its multifaceted mechanism of action, which involves multiple targets. Regarding its anticonvulsant properties, VPA increases the concentration of the inhibitory neurotransmitter gamma-aminobutyric acid (GABA), blocks voltage-sensitive sodium channels, activates calcium-dependent potassium conductance, and reduces excitatory neurotransmission by decreasing NMDA-mediated currents and the release of aspartate [50]. These actions collectively contribute to a reduction in neuronal excitation and an increase in neuronal inhibition.

While VPA exhibits anticonvulsant properties, it has been shown to induce proinflammatory cytokine production in humans [77, 90]. We have recently demonstrated that this proinflammatory effect may be due to VPA’s ability to augment HC activity, particularly those formed by connexin (Cx) 43, Cx30, Cx26, and pannexin-1 (Panx1) [34, 35]. Given the limited research in targeting HC-mediated neuroinflammation to improve epilepsy pharmacology [40, 91], strategies to restore a non-inflamed brain state in epileptic patients are crucial. This could potentially reduce seizure frequency and severity.

Boldo (*Peumus boldus M.*) is a Chilean endemic tree being investigated for its potential as a chronic anti-inflammatory treatment and an adjuvant to ASMs. Its leaves contain alkaloids, flavonoids, phenolic acids, and essential oils conferring antioxidant and anti-inflammatory properties ([24, 25, 73]). Boldine, active alkaloid in Boldo, has shown protective effects in animal inflammatory models [7, 79, 99], potentially through the blockade of Cx and Panx1 HCs [44, 100]. Epilepsy is associated with increased Cx HC activity, which can initiate an inflammatory cascade that exacerbates seizures [59]. Our recent findings show that inhibiting Cx HCs with the selective blocker D4 prevents neuroinflammatory responses, neuronal distress, and epileptic seizures, leading to reduced mortality in a murine model of pilocarpine-induced temporal lobe epilepsy (TLE) [40]. Due to the neuronal loss that occurs in the hilus (polymorphic layer) in TLE, the dentate gyrus (DG) emerges as one of the most relevant regions to evaluate [72]. The DG's physicochemical properties, when dysregulated, can predispose the brain to TLE, often initiating seizures (the epileptic focus). This makes it a crucial area for studying HCs activation. Furthermore, evidence suggests that Panx1 HCs contribute to releasing proinflammatory interleukins and glutamate [95], thereby playing a role in seizure generation [6, 21, 36, 87]. Consistent with this, the absence of Panx1 has been shown to decrease epileptic activity [5]. Therefore, understanding the role of key HCs expressed by glial and neuronal cells in epilepsy is of significant interest, as these membrane channels, along with other non-selective channels, are involved in the pathophysiology of the disease [33].

To explore the therapeutic potential of targeting HCs in epilepsy, we examined their involvement in glial cells and neurons. Thus, we used a chronic epilepsy model, pentylenetetrazole (PTZ)-induced kindling in mice and administered VPA and/or pulverized Boldo leaf. Both treatments achieved 100% survival and significantly ameliorated seizure severity, reversed HCs overactivity in astrocytes and microglia, and the increased astrogliosis observed in the epileptic model. However, only Boldo significantly reduced the exacerbated HCs activity in neurons, and the increased microglial reactivity in the epileptic model. Furthermore, water-soluble compounds from Boldo infusion specifically inhibited Cx43 and Panx1 HCs without affecting Cx43 gap junction channels.

## Materials and methods

### Chemicals

Dulbecco's Modified Eagle's Medium (DMEM), DMEM Nutrient Mixture F12 (DMEM F12), penicillin, streptomycin, geneticin (G418), and fetal bovine serum (FBS) were obtained from Gibco R Invitrogen (Carlsbad, CA, USA). Ethidium bromide (Etd +), valproic acid sodium salt (VPA), and PTZ were purchased from Sigma-Aldrich (Burlington, Massachusetts, USA). 4′, 6-diamidino-2-phenylindole (DAPI) was purchased from TOCRIS Bioscience. Boldo leaves were obtained from Supremo Boldo teabags (Supremo, Valparaíso, Chile).

### The PTZ kindling model of epilepsy

**Fig. 1:**
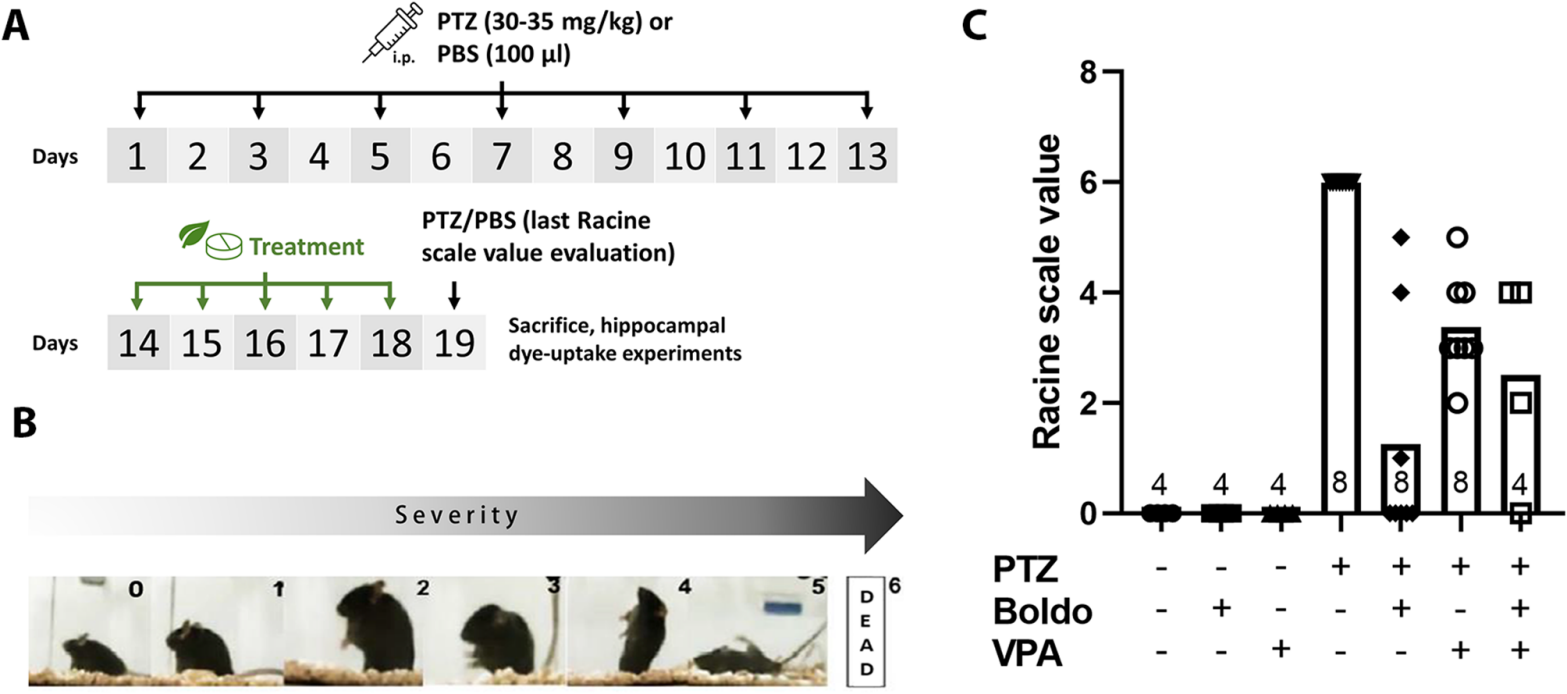
Boldo leaves and/or valproate reduce mortality and seizure severity in chronic pentylenetetrazol-induced epilepsy model. **A** Schematic representation of the chronic epilepsy model. **B** Racine scale for seizure severity classification (adapted from [85]. 0: Normal behavior, no abnormalities; 1: Immobilization, lying face down; 2: Head nodding, myoclonic facial and forelimb or hindlimb spasm; 3: Continuous myoclonic jerks, stiff tail; 4: Lifting on two legs, tonic seizure, lateral fall; 5: Tonic–clonic seizure, falls backward, runs and jumps wildly; 6: Death. **C** Mortality reduction (100% to 0%) and decreased Racine scale scores in epileptic mice treated with Boldo, VPA, or both (*n* = 4). Each point represents an individual mouse's response

Two-month-old male C57BL/6 mice were used in this study. All procedures were approved by the Bioethics Committees for the Care and Use of Laboratory Animals of University of Valparaíso, Chile (Cbc 02/2020 and BEA209-24), and in accordance with the internationally accepted principles for laboratory animal use and care as found in the European Community guidelines (EEC Directive of 1986; 86/609/EEC). To induce epilepsy, mice were administered seven intraperitoneal (i.p.) injections of a subconvulsivant dose of PTZ (30–35 mg/kg) dissolved in phosphate-buffered saline (PBS) every other day, following a previously established protocol [78]. Chronic epilepsy was defined as reaching a Racine scale score of 4 or higher (Fig. 1B).

After epilepsy induction, mice received a five-day treatment consisting of 1 g of peanut butter as the vehicle, administered alone (control) or mixed with either 50 mg/kg/day of Boldo (Supremo leaves), 300 mg/kg/day of VPA, or both. This Boldo dose was chosen based on previous studies demonstrating anticonvulsant efficacy after 7–8 days of boldine administration in PTZ-induced epilepsy model [61] and a published protocol confirming that 50 mg/kg/day dosing via peanut butter is a viable oral delivery method [39]. The five-day treatment period was designed to overlap with the critical phase of PTZ kindling, enabling us to evaluate both seizure susceptibility and neuroinflammatory responses during the development of chronic epilepsy.

Following five days of treatment, mice were re-injected with either PTZ (30–35 mg/kg) or 100 µL PBS. Mice reaching a Racine score of 6, signifying death from intense convulsions, were immediately decapitated. Surviving mice were sacrificed 30 min after the injection via cervical dislocation. The brains were then transferred to oxygenated (95% O_2_/5% CO_2_) artificial cerebrospinal fluid (ACFS, in mM: 25 NaHCO_3_, 25 glucose, 2.5 KCl, 1.25 NaH_2_PO_4_, 2 CaCl_2_, 1 MgCl_2_ and 125 NaCl, pH 7.4).

### Analysis of bioactive molecules present in dried Boldo leaves.

#### Total phenolic compounds (TPC) and total flavonoids (TF)

Two samples of 1 g from commercial Supremo leaf Boldo teabags (Batch number I1 17.02 and G1 03.01), were extracted using 10 mL of 80% methanol in water at 40 °C in an ultrasonic bath for 10 min, then centrifuged at 4 °C for 5 min at 5000 rpm, and the supernatant was separated and saved. This operation was repeated until obtaining 40 mL of supernatant, carrying it to 50 mL with extractive solvent. These extracts were filtered and stored at − 20 °C until use.

The TPC was evaluated as described by Velioglu et al., [89] with the Folin-Ciocalteu reagent, the TF content was determined with the aluminium chloride reagent as described by AL-Ghudani & Hossain [3].

#### Catechin

The high-perfomance liquid chromatography method (HPLC) proposed by Celis-Plá et al. [10] with modifications was used for the quantification of catechin. For the analysis, 2 mL of each extract were filtered through 0.2 µm nylon filters. The analytical process was carried out using a Shimadzu chromatograph with autosampler SIL-20AC, oven column CTO-20AC, quaternary pump LC-20AD, and diode array detector SPD-M20A at 280 nm. An InertSustain C18 (250 mm × 4.6 mm, 5 μm) column was employed with a precolumn CC 8/4 Nucleodur C18 Gravity (4.6 mm, 5 μm). The mobile phase was aqueous phosphoric acid 0.1% v/v (eluent A) and acetonitrile (eluent B), at a flow rate of 1.0 mL/min. The gradient program was as follows: (time, min/%B) 0/90%, 12/88%, 16/88%, 16.01/85%, 25/85%, 25.01/70%, 35/70%, 35.01/65%, 40/65%, 40.01/90%, 50/90%, 50.01/100%, 60/100, and the injection volume was 20 μL. The operational conditions included an oven temperature of 40 °C and detection at a wavelength of 280 nm. A calibration curve was built with concentrations of catechin in the range 50–240 µg/mL.

#### Total alkaloids and boldine

Alkaloids, including boldine, were extracted and quantified using the method described in the Chilean Pharmacopeia (2016).

### Dye uptake and immunofluorescent staining of brain slices

Following brain extraction from mice, they were allowed to stabilize in the vibratome chamber for 10 min in oxygenated ACFS (95% O_2_/5% CO_2_) (Leica VT1200, Wetzlar, Germany). Subsequently, 300 µm thick coronal slices were prepared using the vibratome (cutting speed: 0.40 µm/s). These slices were then incubated in oxygenated ACFS for 1 h at room temperature for stabilization. To assess HC activity via dye uptake, 5 µM Etd + was applied for 10 min [70, 71]. After Etd + incubation, slices were washed 5 times for 10 min with oxygenated PBS and fixed with 4% paraformaldehyde. After 5 additional PBS washes, immunohistochemical staining was performed. Slices were incubated overnight at 4 °C with the following primary antibodies: rabbit anti-GFAP 1:3.000, Antibodies.com A85419) for astrocytes, or rabbit anti-Iba1 (1:3.000, Antibodies.com, A104332) for microglia, and mouse anti-NeuN (1:100, MERCK MAB377) for neurons. The next day, after 5 washes, the sections were incubated for 1 h at 4ºC with secondary antibodies: goat anti-rabbit IgG-Cy2 (1:300, Jackson ImmunoResearch, 111–225-144) and goat anti-mouse IgG-Cy2 (1:300, Jackson ImmunoResearch, 115–225-166). Additionally, slices were washed 3 times with PBS and mounted on glass slides with Fluoromount G DAPI mounting medium. Finally, samples were visualized and acquired in an upright confocal laser-scanning Nikon D-Eclipse C1 confocal microscope (Tokyo, Japan) using a Nikon immersion-oil 40x (N.A 1.30). Single confocal images of the DG region were acquired with EZ-C1 FreeViewer (Nikon Instruments). Seventeen z-stacks were acquired at 1 µm intervals for each experimental condition. These stacks were then volume-rendered into single 2D images using EZ-C1 FreeViewer (Nikon Instruments). Subsequently, the background was subtracted, and channels (red, green, blue) were separated using Nikon NIS Elements Advanced Research software (Nikon Instruments). To quantify the uptake of Etd + , a fixed-size circular region of interest (ROI) was defined in ImageJ and applied to the nuclei of GFP-, Iba1-, or NeuN-positive cells (green/red co-localization). All positive cells for GFAP and Iba1 were evaluated, allowing for the determination of the number of activated astrocytes and microglia. In the case of NeuN, due to the high density of NeuN-positive neurons in the DG *granular layer*, the number of NeuN-positive cells was determined within a fixed-size ROI in the same DG region of all images, and Etd + uptake was measured in a minimum of 10 cells per condition. Finally, the fluorescence intensity of GFAP and Iba1 was measured in the DG hilus using a fixed-size triangular ROI.

### IL-1β, IL-6, TNF-α, and MDA measurements by ELISA

Blood samples were collected via cardiac puncture using syringes pre-coated with heparin. Plasma was separated by centrifugation at 3000 rpm for 15 min and transferred into 1.5 mL microtubes. The samples were then stored at –80 °C until further analysis.

Pro-inflammatory cytokine levels—including IL-1β, IL-6, and TNF-α—were quantified using Mouse Invitrogen ELISA kits (IL-1β: #BMS6002-2; IL-6: #KMC0061; TNF-α: #BMS607-3), following the manufacturer's instructions. Malondialdehyde (MDA), a marker of oxidative stress, was measured using the MDA Colorimetric Assay Kit (Invitrogen, #EEA015), also according to the manufacturer’s protocol.

Cytokine and MDA concentrations were determined by extrapolating from standard curves, with absorbance readings obtained from triplicate measurements. Absorbance was measured using a microplate reader (BioTek 800TS, Agilent, USA). For cytokine assays, absorbance was recorded at 450 nm with correction at 540 nm. For the MDA assay, absorbance was measured at 532 nm. Final concentrations were calculated by subtracting blank values and averaging duplicate readings of standards, controls, and samples. Results were expressed in picograms per milliliter (pg/mL) of plasma for cytokines, and in μmol/L for MDA.

### HeLa cell cultures

HeLa cells stably transfected with mouse connexin43EGFP (HeLa Cx43) or mouse pannexin1EGFP (HeLa Panx1) previously described were used [27, 32, 42]. Cells were cultured in low-glucose DMEM (supplemented with 10% FBS, 50 U/mL penicillin, and streptomycin) and 5 µl/ml of the antibiotic G418 at 37 °C in a humidified incubator under 5% CO_2_/95% O_2_.

### Dye uptake and time-lapse fluorescence imaging

To evaluate HCs function, dye uptake assays were performed on HeLa Cx43, and HeLa Panx1 cells seeded onto 25 mm coverslips. DAPI uptake was measured as previously described [34, 35]. DAPI serves as a permeability trace, providing insights into the functional state of HCs [70, 71]. Cells were incubated in Krebs solution (in mM: 118 NaCl, 4.7 KCl, 3 CaCl_2_, 1.2 MgCl_2_, 10 glucose, 20 HEPES, 9.9 Tris; pH 7.4) and divalent cation-free solution (DCFS, in mM: 118 NaCl, 4.7 KCl, 10 glucose, 20 HEPES, 9.9 Tris; pH 7.4), both supplemented with 5 µM DAPI. DCFS is a standard stimulus for increasing Cx43 HCs opening probability. Alternatively, mechanical stretch (MS), induced by dropping 8 mL of media from 10 cm using a 1000 µL micropipette, [55] stimulates Panx1 HCs opening. For each experiment (*n* = 1, representing ≥ 20 cells from a single culture), cell nuclei fluorescence was measured using a Nikon Eclipse Ti inverted microscope (Japan). Images were acquired every 15 s for 5 min per condition. ROI selection and offline analysis were performed as previously described [34, 35].

### Dye coupling assay

To test the functionality of the formed gap junction channels (GJC), dye transfer experiments with Etd + in HeLa Cx43 cells were performed. HeLa Cx43 cells were cultured on glass coverslips until reaching 80–90% confluence. Cells were visualized using an inverted microscope equipped with a xenon arc lamp and a Nikon B filter (excitation: 450–490 nm, emission: > 520 nm). Single cell was microinjected with Etd + (25 mmol/L), and dye transfer to adjacent cells was observed after 5 min. The incidence of coupling was defined as the percentage of successful injections demonstrating dye spread to more than one adjacent cell. The coupling index was calculated by dividing the mean number of coupled cells by the number of instances where coupling occurred. For each experimental replicate (*n* = 1), a minimum of 10 cells were injected to assess GJC.

### Statistical analysis

All statistical analyses were performed using the GraphPad 8 Prism software (GraphPad Software Inc., La Jolla, CA). Statistical details are provided in each figure legend. First, potential outliers were assessed using the ROUT method [64], which combines robust nonlinear regression with False Discovery Rate control. If an outlier was identified, it was removed from all relevant data. A conservative parameter (Q = 1%) was applied to minimize the likelihood of excluding valid data points, and the main conclusions remained consistent regardless of outlier removal. Following outlier assessment, data normality was tested using Shapiro–Wilk test, and equality of variances was checked with the Brown-Forsythe test. For datasets that passed these tests and with more than two groups, data were analyzed using a one-way ANOVA test followed by a Tukey post-test for multiple comparisons. Because the dye coupling index involved only two groups, these data were analyzed using an unpaired t-test instead of ANOVA. For datasets that did not pass the Shapiro–Wilk test, Kruskal–Wallis test followed by Dunn’s multiple comparison test were performed. All data were presented as mean ± standard error of the mean (SEM). Differences were considered statistically significant at *p* < 0.05.

## Results

### Antiepileptic effect of Boldo leaves and/or valproate on an epileptic animal model

To evaluate the antiepileptic effects of Boldo leaves, both alone or in combination with VPA, we employed the PTZ kindling model in adult C57BL/6 mice. This chronic chemical kindling model, commonly used to study epilepsy pathophysiology [78], involves seven intraperitoneal injections of subconsultant PTZ dose (30–35 mg/kg) administered every other day (Fig. 1A). The development of the epileptic phenotype was assessed using the Racine scale [85] (Fig. 1B). The chosen PTZ concentration aimed to minimize mortality during kindling. After completing the seven injections of PTZ, mice underwent a 5-day treatment period involving Boldo leaves and/or VPA. Subsequently, a final subconvulsant PTZ injection was administered, and mice were monitored for 30 min to assess the Racine scale score.

In the PBS-injected control group (*n* = 4), no mice exhibited seizure activity, consistently scoring 0 on the Racine scale (Fig. 1C). Conversely, untreated epileptic control mice (PTZ + , Boldo -, VPA-, *n* = 8) developed severe seizures reaching a Racine scale score of 6, culminating in mortality. However, a 5-day treatment with Boldo leaves (PTZ + , Boldo + , VPA-, n = 8) significantly protected against severe seizures. Five Boldo-treated mice were seizure-free (Racine scale: 0), while three of them experienced seizures (Racine scale: 1, 4, and 5), followed by recovery, demonstrating an apparent antiepileptic effect. This protective effect of Boldo appeared superior to that of VPA alone. Mice treated with VPA (PTZ + , Boldo -, VPA + , *n* = 8) exhibited seizures (Racine scale: 2–5) after the final PTZ injection, although all mice survived. Combined treatment with Boldo and VPA (PTZ + , Boldo + , VPA + , *n* = 4) also resulted in positive outcomes. One mouse showed no seizures (Racine scale: 0), and three experienced mild to moderate seizures (Racine scale: 2, 4, 4), with all mice surviving.

These data suggest that a 5-day Boldo treatment effectively exerts a significant antiepileptic effect, surpassing the efficacy of VPA alone in this experimental model. The combined Boldo and VPA treatment yielded results comparable to Boldo monotherapy, indicating a potential reversal of the epileptic phenotype and a significant reduction in seizure severity.

To better understand the basis of Boldo’s antiepileptic activity observed in our experimental model, we next analyzed the phytochemical composition of the dried and powdered Boldo leaves used in our treatments. Bioactive compound quantification was performed using HPLC. As shown in Table 1, the analysis revealed the presence of notable amounts of total alkaloids, polyphenols (as gallic acid equivalents), flavonoids (as quercetin equivalents), as well as specific compounds such as boldine and catechin. Two different batches of Boldo teabags from the brand Supremo were tested, both containing consistent levels of these key phytochemicals, though some variability was observed between batches. Importantly, boldine content was quantified by HPLC in both batches, and the values were similar (Table 1).

**Table 1:** Total polyphenols, flavonoids, and alkaloids, boldine and cathechin in powdered Boldo leaves from Supremo teabags. Values represent the mean of three replicate measurements of the same sample ± standard deviation

Bioactive compounds	Batch number Boldo teabags	
	I1 17.02	G1 03.01
Total alkaloids (% DW)	0.125 ± 0.002	0.118 ± 0.003
Boldine (% DW)	0.035 ± 0.004	0.030 ± 0.003
Total polyphenols (mg/g DW)	54.0 ± 0.1	33.0 ± 0.3
Total flavonoids (mg/g DW)	45.0 ± 0.2	32.3 ± 0.1
Catechin (mg/100 g DW)	196.9 ± 0.4	101.4 ± 0.6

### Boldo leaves and valproate reduce dye uptake in hippocampal astrocytes, microglia, and neurons of PTZ kindling mice

Since the opening of both Cx and Panx1 HCs is implicated in neuroinflammation [14] and heighten the membrane permeability of brain cells, we evaluated the membrane permeability in astrocytes, microglia, and neurons from hippocampal slices using Etd + uptake. Etd + , a positively charged dye that fluoresces upon binding to DNA, enters cells through large-pore plasma membrane channels, such as Cx and Panx HCs [70, 71].

**Fig. 2:**
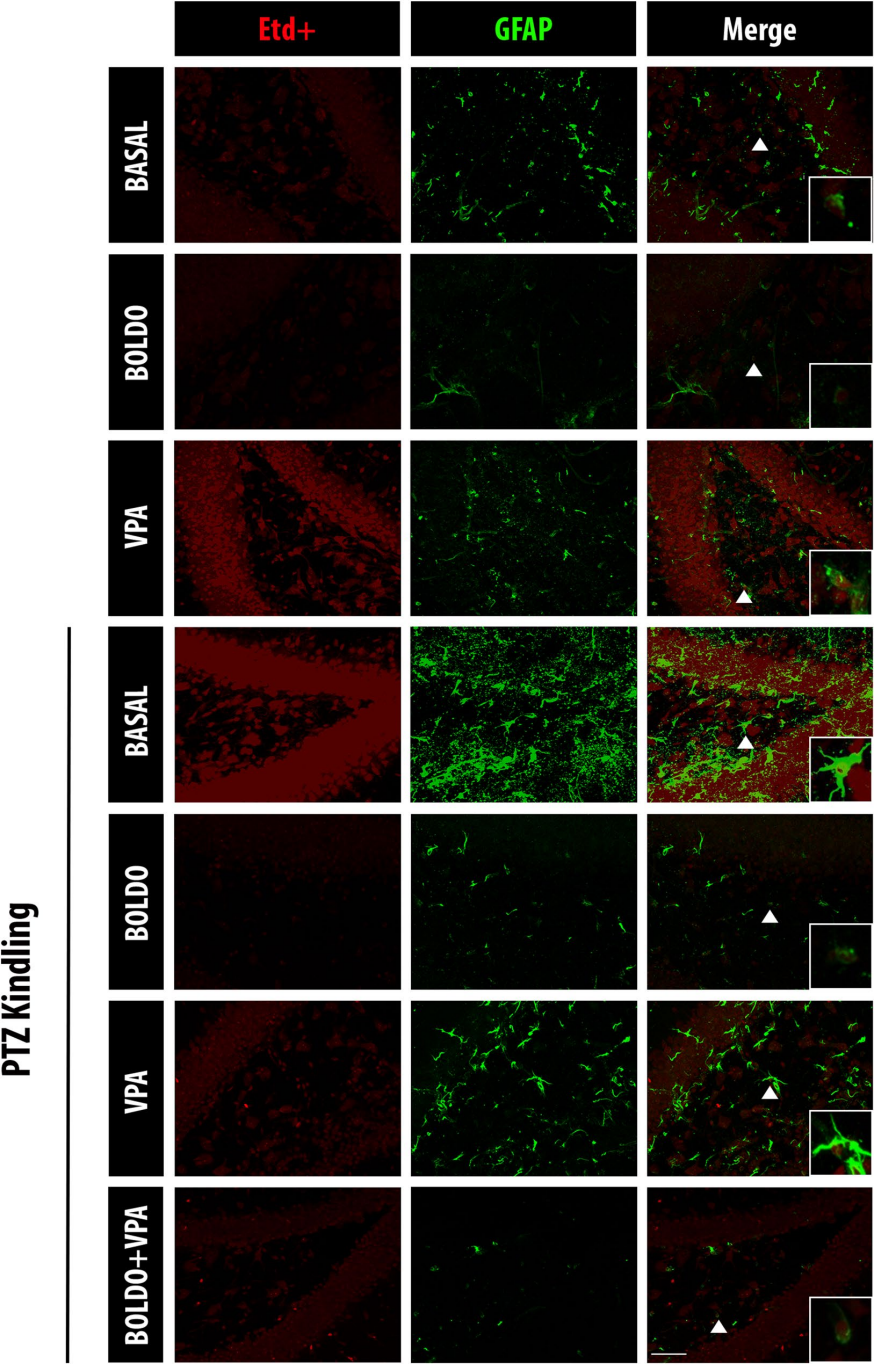
Boldo leaves and/or valproate affect dye uptake in dentate gyrus astrocytes of epileptic mice. Representative confocal images of the DG are shown Etd + fluorescence (red, left), GFAP fluorescence (green, middle), and the merged Etd + /GFAP (right). An arrowhead in the merged panel (bottom right) highlights an enlarged astrocyte. Scale bar = 50 µm. *n* = 4 for non-epileptic, PTZ + , Boldo + , and VPA + groups; *n* = 8 for all other groups. *n* represents individual mice

**Fig. 3:**
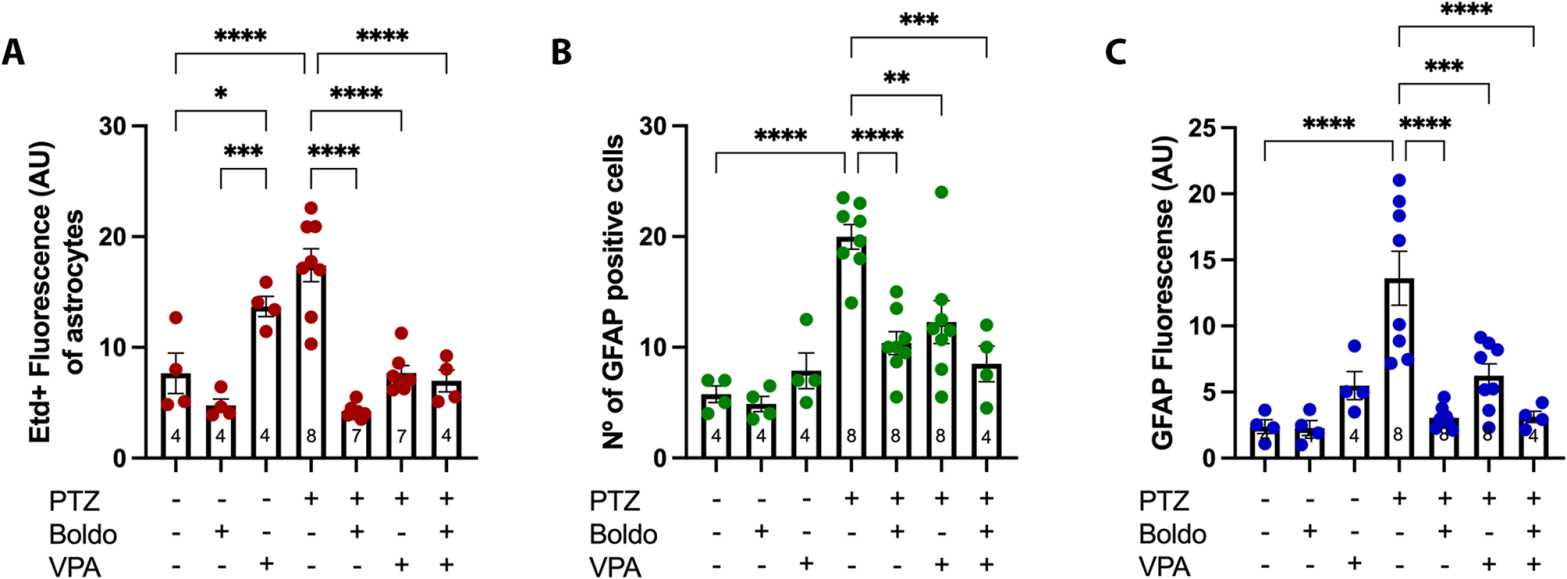
Dye uptake and GFAP reactivity of astrocytes under different conditions. **A** Quantification Etd + fluorescence intensity in DG astrocytes (GFAP), representing HCs activity. **B** Number of GFAP-positive cells astrocytes in DG. **C** GFAP fluorescence intensity within the DG, indicating astrocyte reactivity. Data points represent the sample size (*n*) for each condition, each *n* from a separate animal (*n* = 4 for non-epileptic mice and the PTZ + , Boldo + , and VPA + conditions. For all other conditions, *n* = 8, except for the PTZ + , Boldo + , VPA- and PTZ + , Boldo -, and VPA + conditions in panel A, where *n* = 7 due to the removal of an outlier identified using the ROUT method.). Shapiro–Wilk test, a one-way ANOVA followed by Tukey's post hoc test were performed (****P < 0.0001, ***P < 0.001, **P < 0.01, *P < 0.05). ANOVA summary reports: (A) F (6, 31) = 22.44, *p*-value: < 0.0001, *p*-value summary: ****,* R*2: 0.8128; (B) F (6, 33) = 13.12,* p*-value: < 0.0001,* p*-value summary: ****,* R*2: 0.7046; (C) F (6, 33) = 12,16,* p*-value: < 0.0001,*p*-value summary: ****,* R*2: 0,6886

We found that astrocytes in the DG of these mice exhibited varying Etd + fluorescence intensity that depends on the treatments (Fig. 2). Quantification of Etd + fluorescence intensity in DG astrocytes (Fig. 3A) showed that Boldo leaf treatment in non-epileptic animals did not significantly alter dye uptake compared to the control. In contrast, VPA treatment significantly increased Etd + uptake. This finding aligns with our previous observations that VPA upregulates the activity of Cx43, Cx30, Cx26, and Panx1 HCs [34, 35]. Similarly, PTZ alone (PTZ + , Boldo -, VPA-) also significantly increased dye uptake. Notably, Boldo alone, VPA alone, or the combined Boldo+VPA treatment significantly reduced Etd + uptake in astrocytes from epileptic animals.

GFAP is the principal intermediate filament in mature astrocytes of the central nervous system (CNS). Its synthesis is known to rapidly increase during astrogliosis [23], making elevated GFAP protein content or immunostaining a reliable marker of astrogliosis. We assessed astrogliosis by quantifying the number of GFAP-positive cells and the intensity of GFAP fluorescence. The epileptic control mice (PTZ + , Boldo -, VPA-) exhibited a significant increase in GFAP-positive cells (Fig. 3B) consistent with astrogliosis. In contrast, epileptic mice treated with either Boldo leaves, VPA, or the combined therapy showed a significant reduction in GFAP-positive cells, suggesting a reversal of the astrogliosis process. Notably, the most pronounced reduction was observed in mice treated with Boldo alone. Analysis of GFAP fluorescence intensity levels also revealed that VPA treatment in non-epileptic control mice (PTZ-, Boldo -, VPA +) resulted in increased fluorescence compared to untreated controls (Fig. 3C), indicating that VPA itself induces astroglial activation. Furthermore, the epileptic control group exhibited significantly elevated GFAP fluorescence, which was effectively attenuated by treatment with Boldo leaves and/or VPA.

**Fig. 4:**
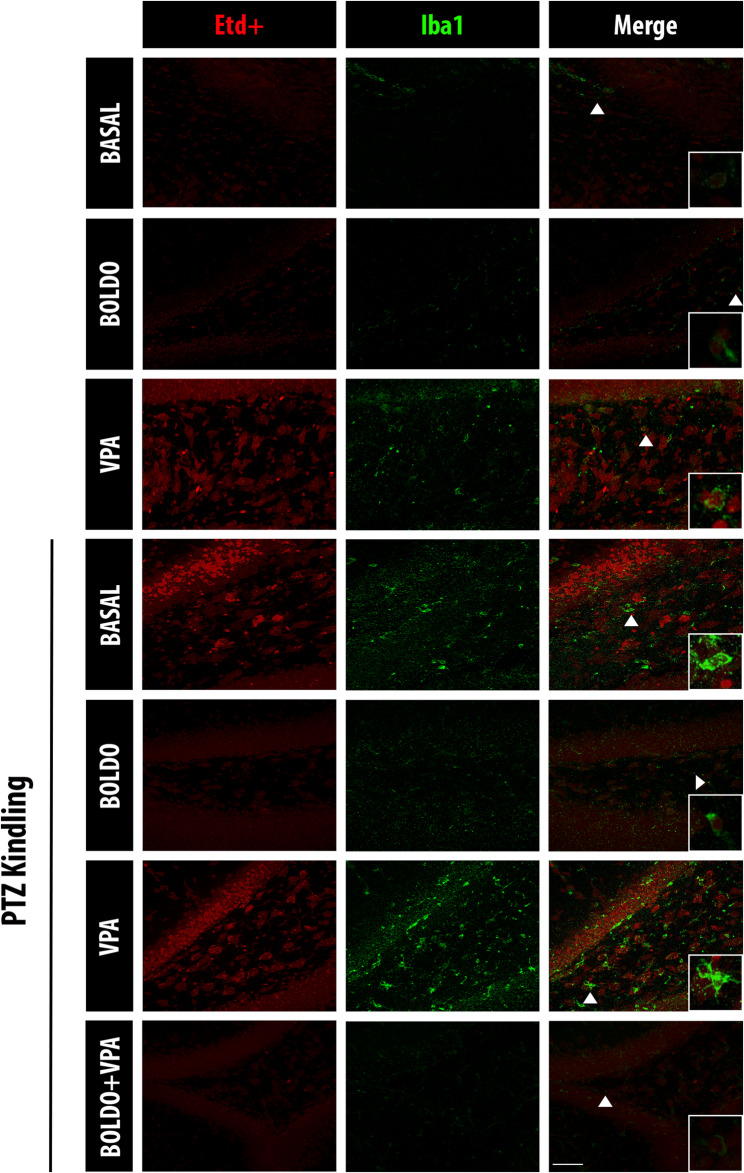
Boldo leaves and/or valproate affect dye uptake in dentate gyrus microglia of epileptic mice. Representative confocal images of the DG are shown. Etd + fluorescence (red, left), Iba1 fluorescence (green, middle), and the merged Etd + /Iba1 (right). The arrowhead in the lower right corner of the merged image highlights an example of increased microglial cell body size. Scale bar = 50 µm. Sample sizes: *n* = 4 for non-epileptic mice (all PTZ- conditions), PTZ + , Boldo + , and VPA + ; *n* = 8 for all remaining conditions. *n* represents individual mice

**Fig. 5:**
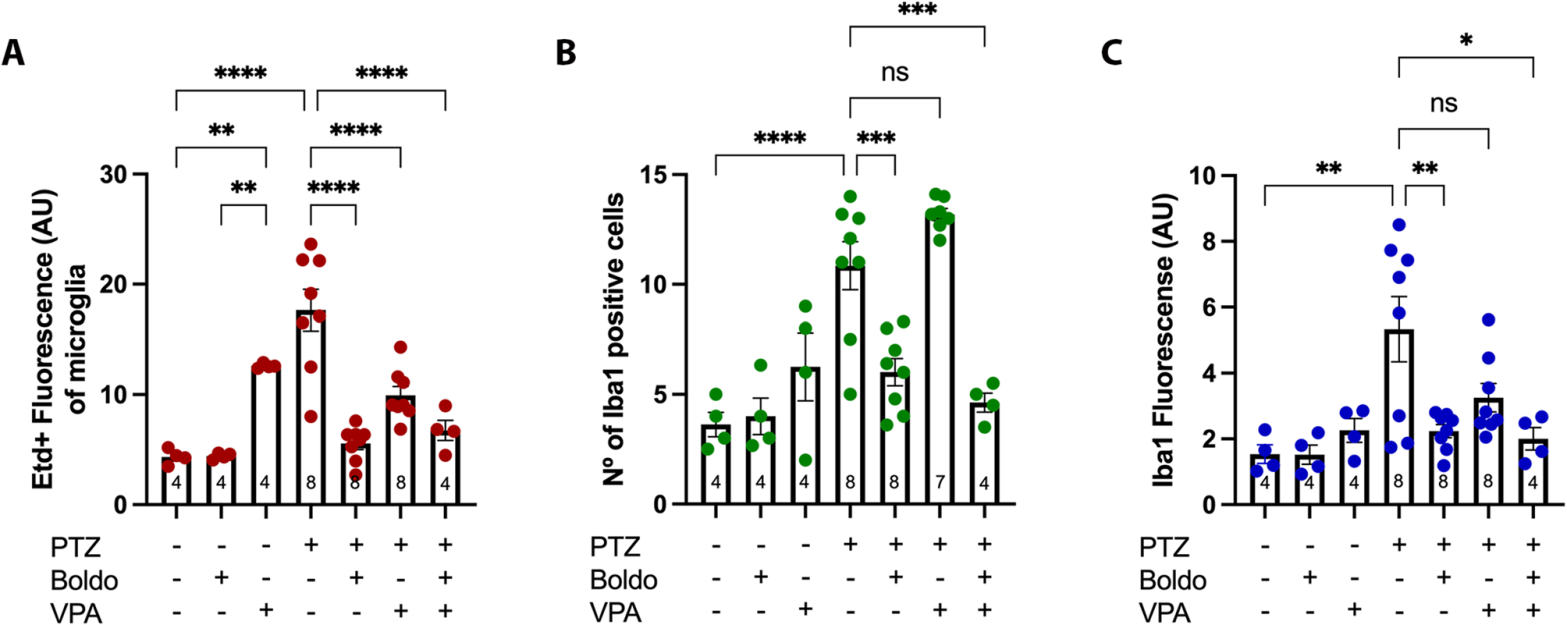
Dye uptake and Iba1 reactivity of microglia under different conditions. **A** Quantification of Etd + the fluorescence intensity in DG microglia (Iba1), representing HCs activity. **B** Number of Iba1-positive cells microglia in DG. **C** Iba1 fluorescence intensity within the DG, indicating microglia reactivity. Data points represent the sample size (*n*) for each condition, each *n* from a separate animal (*n* was 4 for non-epileptic mice and the PTZ + , Boldo + , and VPA + conditions. For all other conditions, *n* was 8, except for the PTZ + , Boldo -, and VPA + condition in panel B, where *n* was 7 due to removing an outlier identified using the ROUT method.). Shapiro–Wilk test, a one-way ANOVA followed by Tukey's post hoc test were performed (****P < 0.0001, ***P < 0.001, **P < 0.01, *P < 0.05). ANOVA summary reports: (A) F (6, 33) = 19.36, *p*-value: < 0.0001, *p*-value summary: ****,* R*2: 0.7787; (B) F (6, 32) = 19.00,* p*-value: < 0.0001,* p*-value summary: ****,* R*2: 0.7808; (C) F (6, 33) = 5,468,* p*-value: 0.0003, p-value summary: ***,* R*2: 0,4985

A comparable pattern of Etd + uptake was observed in microglia (Fig. 4). Specifically, microglia from control mice exposed to VPA and from epileptic control mice displayed a significant increase in Etd + uptake (Fig. 5A). This elevated uptake was significantly attenuated in all treated epileptic animals, including those receiving Boldo alone, VPA alone, or the combined therapy (Boldo + VPA). We then evaluated microglial activation by measuring the number of Iba1-positive cells and the immunofluorescence intensity of Iba1. Iba1 is an intracellular protein crucial for microglial cytoskeleton remodeling, and phagocytic activity [49], and it is a widely recognized marker of microglial activation [31, 47, 101]. We found a significantly higher number of Iba1-positive cells and level of Iba1 fluorescence in epileptic mice compared to control mice. Notably, treatment with Boldo, either alone or in conjunction with VPA, significantly decreased the values observed in non-treated epileptic mice, whereas VPA alone did not (Fig. 5B, 5).

**Fig. 6:**
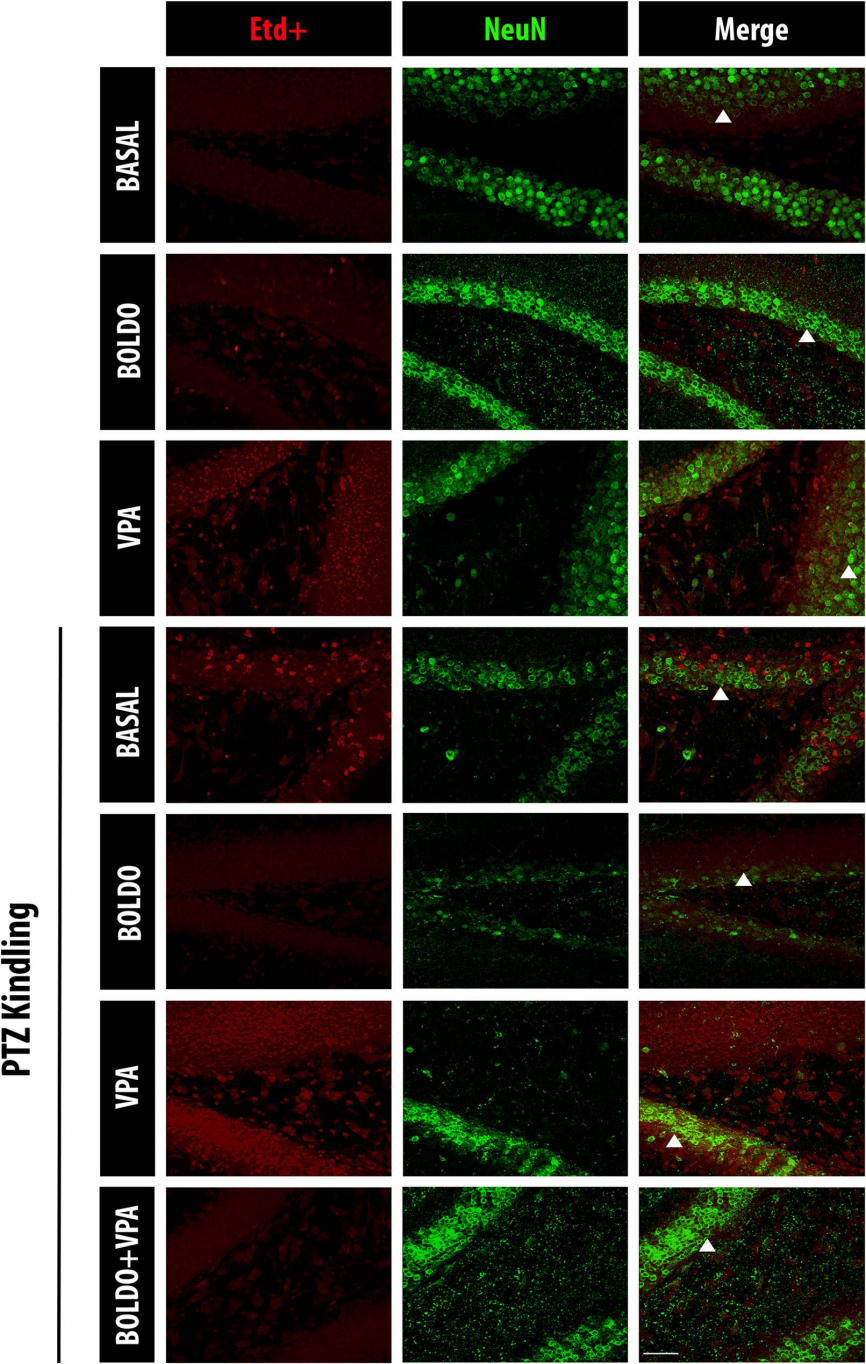
Boldo leaves and/or valproate affect dye uptake in dentate gyrus neurons of epileptic mice. Representative confocal images of the DG are shown. Etd + fluorescence (red, left), NeuN fluorescence (green, middle), and merged Etd + /NeuN (right). The arrowhead in the lower right corner of the merged image highlights an example of increased neuron cell body size. Scale bar = 50 µm. Sample sizes: *n* = 4 for non-epileptic mice (all PTZ- conditions), PTZ + , Boldo + , and VPA + ; *n* = 8 for all remaining conditions. *n* represents individual mice

**Fig. 7:**
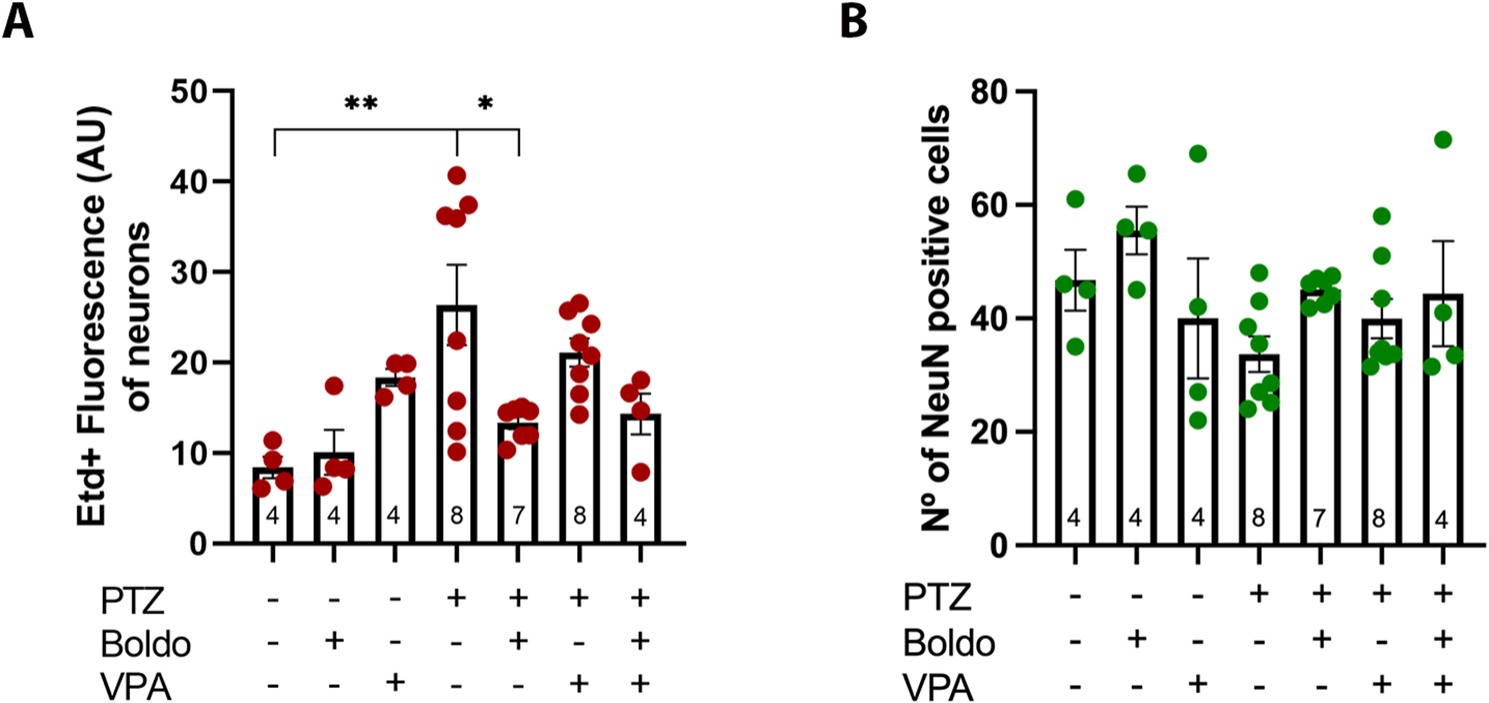
Dye uptake and NeuN reactivity of neurons under different conditions. **A** Quantification of Etd + the fluorescence intensity in DG neuron (NeuN), representing HCs activity. **B** Number of NeuN-positive cells neurons in a determined ROI in DG granular cell layer. Data points represent the sample size (*n*) for each condition, each *n* from a separate animal (*n* was 4 for non-epileptic mice and the PTZ + , Boldo + , and VPA + conditions. For all other conditions,* n* was 8, except for the PTZ + , Boldo + , and VPA- condition, where *n* was 7 due to removing an outlier identified using the ROUT method, in both panels). In panel A, Shapiro–Wilk test, a one-way ANOVA followed by Tukey's post hoc test were performed. ANOVA summary reports: F (6, 32) = 5.454, *p*-value: 0.0006, *p*-value summary: ***,* R*.^2^: 0.5056. In panel B, after Shapiro–Wilk test, Kruskal–Wallis test followed by Dunn’s multiple comparison test were performed. Kruskal–Wallis test (ANOVA results): *p*-value: 0.0790, *p*-value summary: ns; H statistic: 11.32

In addition, the intensity of Etd + fluorescence was quantified in granule cell neurons of the DG under different experimental conditions (Fig. 6). Compared to non-epileptic control mice (PTZ-, Boldo -, VPA-), epileptic mice (PTZ + , Boldo -, VPA-) showed a significant increase in dye uptake, which was significantly reduced upon treatment with Boldo alone. However, VPA treatment did not produce a significant decrease in dye uptake compared to the epileptic control group (Fig. 7A). Regarding the number of NeuN positive cells, which indicate neuronal survival, no significative changes were observed, but a tendency contrary to the Etd + uptake was seen, suggesting less neuronal survival in PTZ treated mice and more survival when they also received Boldo (Fig. 7B). These results imply that Boldo 's neuronal effects are likely mediated through HCs, specifically Panx1 HCs, which are predominantly expressed in these cells [102].

These results generally indicate heightened activation of astrocyte and microglia in epilepsy, along with increased HCs activity within these cell types. Neuronal HCs also appear more active. Notably, treatment with Boldo leaf significantly reduced seizure frequency and improved survival in mice (Fig. 1), highlighting its potential therapeutic value in epilepsy.

To determine whether the reduction in seizure severity due to Boldo leaves and/or VPA treatment (Fig. 1C) is due to the blockade of HCs present in astrocytes and microglia, we can observe the individual values for each sample (*n*) in the different conditions for hippocampal neurons, as well as decreased astrogliosis and microglia reactivity (Table 2). In general, for higher Racine scale values (in the PTZ model), there are higher values in the other variables. These results generally show a relationship between seizure severity, HC activity, and astroglia and microglial activation (Table 2).

**Table 2:** Values for each sample for Racine scale, fluorescence levels, and number of activated cells for each condition. For each sample size (*n*), the Racine scale value (*Racine scale*), Etd + fluorescence (AU) of astrocytes (*Etd* + *fluo. astrocytes*), GFAP fluorescence (AU) (*GFAP fluo.*), number of activated astrocytes (*Nº of astrocytes*), Etd + fluorescence (AU) of microglia (*Etd* + *fluo. microglia*), Iba1 fluorescence (AU) (*Iba1 fluo.*), number of activated microglia (*Nº of microglia*), Etd + fluorescence (AU) of neurons (*Etd* + *fluo. neurons*), and number of NeuN positive cells in a determined ROI in the granular layer (*Nº of neurons*) are shown. Values with an asterisk represent outliers (ROUT method) that were removed before statistical analysis

Basal									
N	Racine Scale	Etd + fluo. astrocytes	GFAP fluo	Nº of astrocytes	Etd + fluo. microglia	Iba1 fluo	Nº of microglia neurons	Etd + fluo	Nº of neurons
1	0	12.686	3.635	7	4.459	s1.65	2.5	9.273	61
2	0	4.828	2.522	4	4.263	1.018	5	11.379	35
3	0	8.017	2.303	5	3.474	2.279	4	6.879	45
4	0	5.120	1.104	7	5.182	1.199	3	6.106	46
Boldo									
N	Racine Scale	Etd + fluo. astrocytes	GFAP fluo	Nº of astrocytes	Etd + fluo. microglia	Iba1 fluo	Nº of microglia	Etd + fluo neurons	Nº of neurons
1	0	4.062	3.687	4	4.348	1.164	2.666	17.426	56
2	0	4.649	2.488	3.5	4.698	0.93	3	6.321	55.5
3	0	6.432	1.914	6.5	4.578	1.806	4	8.383	65.5
4	0	3.917	0.983	5.5	4.070	2.187	6.333	8.236	45
VPA									
N	Racine Scale	Etd + fluo. astrocytes	GFAP fluo	Nº of astrocytes	Etd + fluo. microglia	Iba1 fluo	Nº of microglia	Etd + fluo neurons	Nº of neurons
1	0	15.876	8.495	12.5	12.909	2.795	6	19.913	42
2	0	13.418	5.022	7	12.537	1.316	2	16.169	22
3	0	14.063	4.963	7	12.390	2.070	8	17.493	27
4	0	11.439	3.499	5	12.582	2.853	9	19.896	69
Basal PTZ									
N	Racine Scale	Etd + fluo. astrocytes	GFAP fluo	Nº of astrocytes	Etd + fluo. microglia	Iba1 fluo	Nº of microglia	Etd + fluo neurons	Nº of neurons
1	6	17.011	10.108	18.5	17.116	2.701	7.5	22.418	38.5
2	6	12.725	7.478	18	16.509	1.747	5	15.786	35.5
3	6	17.760	8.863	23	12.511	5.818	11	12.441	48
4	6	10.306	7.187	14	8.019	1.870	11	10.160	43
5	6	17.166	19.419	23.5	19.156	7.426	14	40.700	27
6	6	20.916	16.476	21.8	23.643	8.500	13.2	37.457	25.2
7	6	20.890	18.340	21.4	22.129	6.900	13	36.231	28.6
8	6	22.594	21.041	22.205	22.205	7.730	12.1	35.950	24
Boldo PTZ									
N	Racine Scale	Etd + fluo. astrocytes	GFAP fluo	Nº of astrocytes	Etd + fluo. microglia	Iba1 fluo	Nº of microglia	Etd + fluo neurons	Nº of neurons
1	5	5.518	2.261	15	6.367	1.678	7	15.110	47.5
2	4	9.431*	4.620	13.5	6.353	2.041	6	11.930	44
3	0	3.507	2.948	10	2.724	1.184	4	5.240*	42.5
4	0	4.021	2.117	5.5	3.953	2.635	8	10.369	28*
5	0	4.500	2.831	10.8	6.386	2.563	4.8	14.825	41.8
6	1	3.983	3.154	9.5	5.263	2.805	3.6	11.982	47
7	0	3.879	2.734	10	7.604	2.744	6.4	14.489	46.1
8	0	4.201	3.777	8.7	5.897	2.258	8.3	14.626	46.6
VPA PTZ									
N	Racine Scale	Etd + fluo. astrocytes	GFAP fluo	Nº of astrocytes	Etd + fluo. microglia	Iba1 fluo	Nº of microglia	Etd + fluo neurons	Nº of neurons
1	5	11.293	5.172	24	11.117	5.620	14	22.187	43.5
2	4	8.576	3.617	8	14.309	2.445	12	16.525	31.5
3	4	15.665*	5.237	11.5	8.911	2.058	13	18.750	51
4	2	7.043	2.311	5.5	6.869	2.823	4*	14.263	58
5	3	6.166	9.142	12.5	8.536	2.488	13.2	20.763	34.7
6	3	6.260	8.194	10.7	9.033	4.459	13.3	24.280	33.7
7	3	7.031	7.619	11.7	9.074	2.579	12.7	25.728	34
8	3	7.406	8.693	14.3	11.608	3.555	14.1	26.551	33.3
Boldo + VPA PTZ									
N	Racine Scale	Etd + fluo. astrocytes	GFAP fluo	Nº of astrocytes	Etd + fluo. microglia	Iba1 fluo	Nº of microglia	Etd + fluo neurons	Nº of neurons
1	0	5.530	2.146	7.5	4.498	1.244	3.5	7.913	31.5
2	4	9.237	3.249	10	6.850	2.479	5.5	14.670	41
3	2	8.037	4.203	12	8.996	1.622	4.5	18.051	71.5
4	4	5.113	2.931	4.5	6.652	2.670	5	16.640	33.5

### Boldo treatment reverses PTZ-induced elevation of proinflammatory cytokines and oxidative stress marker MDA in epileptic mice

**Fig. 8:**
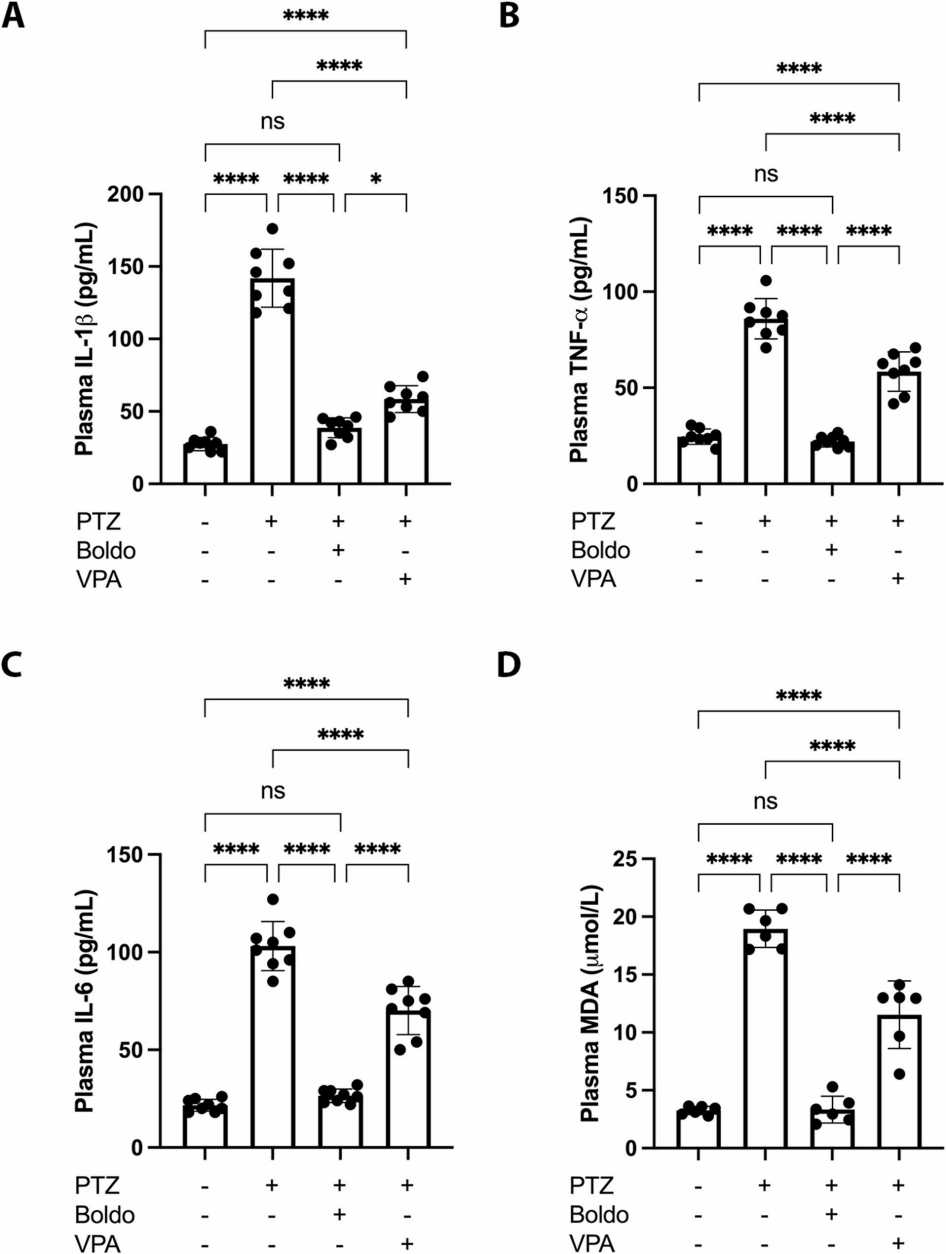
Boldo and valproate modulate blood proinflammatory cytokines and the oxidative stress marker malondialdehyde (MDA). Plasma levels of IL-1β, TNF-α, IL-6, and MDA (a, b, c, and d, respectively) in each condition: non-epileptic control, epileptic control, and epileptic mice treated with Boldo or VPA. Data points represent the sample size (*n*) for each condition, each *n* from a separate animal (*n* = 8 in a, b, and c; and *n* = 6 in d*)*. After the ROUT method, Shapiro–Wilk test was performed confirming normality, and a one-way ANOVA followed by Tukey's post hoc test was performed (****P < 0.0001). ANOVA summary reports: (A) F (3,28) = 155.4, *p*-value: < 0.0001, *p*-value summary: ****,* R*2: 0,9433; (B) F (3, 28) = 123.6, p-value: < 0.0001,* p*-value summary: ****,* R*2: 0,9298; (C) F (3, 28) = 144,0, *p*-value: < 0.0001,* p*-value summary: ****,* R*2: 0,9391; (D) F (3, 20) = 108,2,* p*-value: < 0.0001,* p*-value summary: ****,* R*2: 0,9420

The anti-inflammatory and antioxidant effects of Boldo and VPA were evaluated by measuring plasma concentrations of IL-1β, IL-6, TNF-α (as markers of inflammation), and malondialdehyde (MDA, as a marker of oxidative stress) using ELISA. Four experimental groups were analyzed: control, PTZ-kindling (epileptic) mice, and PTZ-kindling mice treated with either Boldo or VPA. The PTZ-kindling model led to significant increases in IL-1β, IL-6, TNF-α, and MDA levels compared to the control group, indicating heightened inflammatory and oxidative responses. Treatment with either Boldo or VPA significantly reduced these markers compared to untreated PTZ-kindling mice. Notably, Boldo treatment was more effective, as IL-1β, IL-6, TNF-α, and MDA levels in Boldo -treated PTZ-kindled mice were not significantly different from control values. In contrast, while VPA also attenuated these elevations, cytokine and MDA levels in VPA-treated mice remained significantly higher than those in controls. These findings suggest that Boldo exerts a stronger anti-inflammatory and antioxidant effect than VPA in the PTZ-kindling model of epilepsy (Fig. 8).

### Valproate-induced activity of connexin 43 hemichannels is reversed by Boldo leaves infusion, which does not affect the gap junctions formed by connexin 43

**Fig. 9:**
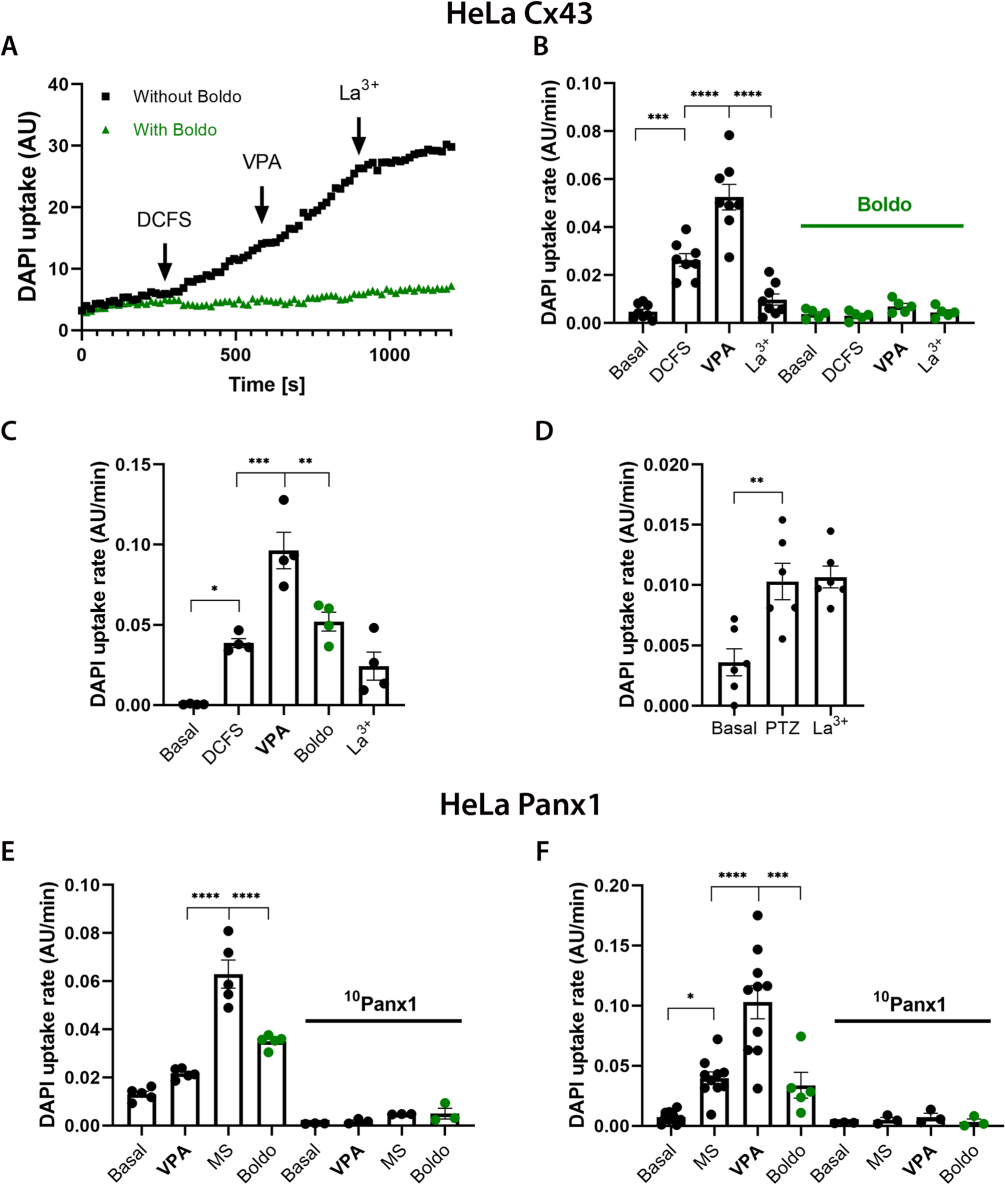
Valproate enhances dye uptake in connexin43/pannexin1-transfected HeLa cells; Boldo infusion prevents and inhibits it. **A** Time course of DAPI fluorescence intensity in HeLa cells expressing Cx43 under basal conditions and after pre-activation with DCFS (600 μM VPA and 200 μM La^3+^), with or without Boldo infusion (Supremo, 0.768 g/L). Corresponding DAPI uptake rates (slopes) are shown in lower graphs. **B** (left) Effect of VPA on Cx43 HCs activity following DCFS pre-activation (*n* = 8). (right) Effect of VPA in the presence of Boldo infusion (*n* = 5). **C** Acute effect of Boldo infusion on HC activity after activation by DCFS and VPA was evaluated (*n* = 4). **D** Effect of 10 mM PTZ on HeLa Cx43 cells under basal conditions (*n* = 6). **E**–**F** Acute effects of Boldo infusion (5 min) on Panx1 HCs. **E** Panx1 HCs were exposed to VPA, mechanical stretch (MS), and Boldo (left: *n* = 5, right (^10^Panx1):* n* = 3). **F** Panx1 HCs were exposed to MS, VPA, and Boldo (left: *n* = 5, right (^10^Panx1):* n* = 3). Experiments were repeated with 200 μM ^10^Panx1, a selective Panx1 HC blocker. Data correspond to mean ± SEM, One-way ANOVA and a Tukey post-test (****P < 0.0001, ***P < 0.001, **P < 0.01, *P < 0.05). Circles represent individual replicates (n), each based on the average response of ≥ 20 cells from one culture. ANOVA results: (B, left) F (3, 28) = 43.92, *p*-value: < 0.0001, ****,* R*^2^: 0.8247; (B, right) F (3, 16) = 2.934, *p*-value: 0.0652, ns,* R*^2^: 0.3549; (C) F (4, 15) = 25.78, *p*-value: < 0.0001, ****,* R*^2^: 0.8730; (D) F (2, 15) = 10.74, *p*-value: 0.0013, **,* R*^2^: 0.5889; (E, left) F (3, 16) = 64.39, *p* value: < 0.0001, ****,* R*^2^: 0.9235; (E, right) F (3, 8) = 0.8824, *p* value: 0.4900, ns,* R*^2^: 0.2486; (F, left) F (3, 16) = 50.14, *p*-value: < 0.0001, ****,* R*^2^: 0.9039; (F, right) F (3, 8) = 3.318, *p*-value: 0.0777, ns,* R*.^2^: 0.5544)

Considering the presence of boldine in Boldo leaves and antiepileptic and HCs blocking properties of Boldo in the chronic epileptic model (Figs. 1–7), we aimed to determine if Boldo infusion (0.768 g/L) could also prevent and reverse the increased activity of HCs. To achieve this, we conducted experiments using HeLa cells expressing Cx43 or Panx1; we induced HCs activity with 600 µM VPA, as previously described [34, 35], and then applied Boldo infusion (0.768 g/L) (Fig. 9).

In HeLa Cx43 cells, Boldo infusion prevented (Fig. 9A and 9B) and acutely reversed the activation of Cx43 HCs induced by VPA (Fig. 9C). Furthermore, PTZ also acutely increased the HCs activity (Fig. 9D), consistent with observations in hippocampal slices (Figs. 2–7). Similarly, Boldo infusion rapidly blocked Panx1 HCs activity induced by mechanical stretch (MS) (Fig. 9E) or VPA (Fig. 9F), with significant inhibition observed after only 5 min. These results suggest that Boldo exerts its effects by blocking Cx43 and Panx1 HCs (Fig. 9), which likely contributes to the reduction of inflammation (Figs. 2–7) and the mitigation of epileptic symptoms (Fig. 1) in an animal model of epilepsy.

Previous research has demonstrated that boldine blocks Cx43 and Panx1 HCs but does not block GJCs formed by Cx43 [44, 100]. We found that Boldo infusion has a similar effect as pure boldine by blocking Cx43, and Panx1 HCs (Fig. 9). To confirm that Boldo infusion also did not affect the activity of Cx43 GJCs, we assessed intercellular dye transfer. Boldo infusion at 0.768 g/L, the concentration that blocked HCs, did not alter the coupling index, which reflects the extent of intercellular dye transfer, or the incidence percentage of coupling events (Fig. S1C, D).

These results indicate that Boldo infusion, which contains negligible water-soluble boldine [29, 81], exhibits functional properties akin to pure boldine: it inhibits Cx43 and Panx1 HCs (Fig. 9) without affecting Cx43 GJCs (Fig. S1).

## Discussion

Previous evidence shows that blocking HCs can mitigate neuroinflammation and alleviate epileptic symptoms [40, 61, 91]. Given Boldo’s established antioxidant and anti-inflammatory capabilities [24, 25, 73], we explored its potential antiepileptic and anti-inflammatory effects in a murine model of chronic epilepsy.

Our findings demonstrate that PTZ-induced epilepsy significantly elevates HCs activity in hippocampal astrocytes and microglia, with a similar trend observed in neurons, consistent with previous reports [40, 91]. Furthermore, we observed increased reactivity of these cells. In alignment with prior studies, astrocytes displayed elevated GFAP levels and increased number of reactive astrocytes, providing significant evidence of astrogliosis [8, 11, 17, 40]. Microglia activation also showed a similar trend, with an increased number of Iba1 positive cells and Iba1 fluorescence levels, reflecting an increased microgliosis in epileptic mice compared with controls. These data support the established literature, which consistently reports increased neuroinflammation in the PTZ kindling model [46, 51, 52, 68, 74, 80, 92, 93]. Regarding neuronal survival, a protective trend with Boldo treatment was observed in the number of NeuN-positive cells, a commonly used neuronal survival marker [45, 82, 88, 98]. However, it should be noted that reduced NeuN labeling may also reflect protein depletion or loss of antigenicity, rather than neuronal death alone [86].

Also, our model effectively replicates temporal lobe epilepsy (TLE) features as described for PTZ-induced kindling [19].

Administration of both VPA and/or powdered Boldo leaves for five days completely prevented mortality in epileptic mice subjected to PTZ-induced seizures. This finding highlights Boldo 's significant protective capacity against seizure-related death, a major concern in epilepsy [15, 16, 57], mirroring the efficacy of VPA, a commonly prescribed antiepileptic medication. Furthermore, Boldo treatment alone exhibited a superior ability to mitigate seizure severity compared to VPA alone, implying a distinct and effective anticonvulsant action.

To explore the mechanisms underlying this neuroprotective effect of Boldo, we analyzed its influence on hippocampal cellular activity. Specifically, we assessed astrocytes, microglia, and neurons. Boldo exhibited sufficient efficacy in reversing the astrocyte and microglial reactivity induced by epilepsy. Specifically, astrogliosis was significantly reduced in mice treated with a combination of Boldo and VPA. A similar reduction in microglial activation was also observed, highlighting Boldo 's inherent anti-inflammatory capacity. Notably, VPA treatment alone did not significantly reverse glial cell activation. In contrast, control mice receiving VPA showed a trend to increase astro- and microglial activation markers (nº of activated cells and fluorescence intensity of GFAP and Iba1, respectively), accompanied by a significant increase of Etd + uptake in both astrocytes and microglia, indicating a potential proinflammatory effect of VPA. The literature presents a complex picture of VPA's inflammatory role. Some studies have documented its anti-inflammatory properties in animal models [46, 65, 97]. However, our findings, alongside other reports, suggest that VPA can induce proinflammatory effects, not only in animal models [43] but also in human epileptic patients [77, 90]. Furthermore, while other ASMs, such as vinpocetine and carbamazepine, have been shown to reduce proinflammatory interleukin levels in the rat hippocampus, VPA does not [37]. Our results in the mouse hippocampus further support the notion that VPA has a proinflammatory effect, which might be attributed to its ability to activate HCs, thereby promoting glial dye uptake and neuroinflammation [13, 34, 35]. In contrast, Boldo exerts inhibitory effects on HCs, which may explain its potent anti-inflammatory action [13, 34, 35].

The contradictory reports on VPA's pro- or anti-inflammatory effects likely arise from its dual mechanisms of action. VPA's established antiepileptic function, primarily achieved by modulating GABA neurotransmission in neuronal targets, reduces seizure burden and, consequently, indirectly lessens neuroinflammation associated with epilepsy. Indeed, our data showed that VPA treatment exhibited a similar trend in reducing blood cytokine levels and astrogliosis in the DG of epileptic mice. This suggests that depending on the context, VPA possesses anti-inflammatory properties. The proinflammatory effect of PTZ might be explained by the predominance of excitatory neurotransmission because glutamate can activate microglia [20] and reactive microglia cells release proinflammatory cytokines that activate astrocytes [67]. VPA could inhibit these cell–cell responses by reducing the neuronal excitability [50] and therefore it could reduce the neuroinflammatory response and dye uptake in glial cells triggered by PTZ. On the other hand, the increase in dye uptake of glial cells observed in the hippocampus of control mice treated only with VPA might be partially explained by direct activation of HCs induced by this compound [34, 35]. Taken together, these dual and sometimes opposing effects suggest a mechanistic basis for the lack of synergy in the combination therapy: while VPA reduces neuronal excitability, it simultaneously promotes glial HC opening and inflammation, thereby counteracting Boldo’s HC-inhibitory and anti-inflammatory actions. This antagonistic interaction may explain why Boldo alone appeared more effective than when combined with VPA. Finally, although the number of animals in the combination group (n = 4) was lower than in the single-treatment groups (n = 8), this limitation reflects our effort to follow ethical guidelines, since generating the chronic epilepsy model involves significant animal suffering. Future studies with larger sample sizes, while respecting ethical constraints, will be needed to fully confirm these findings.

PTZ-kindling significantly elevated plasma levels of the proinflammatory cytokines IL-1β, IL-6, and TNF-α, as well as malondialdehyde (MDA), a key marker of lipid peroxidation and oxidative stress [66]. These findings confirm that the epileptic state induced by PTZ is associated with both systemic inflammation and oxidative stress. Treatment with Boldo effectively reversed these alterations, bringing cytokine and MDA levels back to values statistically indistinguishable from those of control animals. This suggests that Boldo exerts a robust anti-inflammatory and antioxidant effect. In contrast, while VPA also significantly reduced the elevated levels of IL-1β, IL-6, TNF-α, and MDA compared to untreated epileptic controls, these markers remained significantly higher than in non-epileptic control animals, indicating not as much mitigation as seen with Boldo treatment. The particularly strong suppression of IL-6 by Boldo is noteworthy, as this cytokine is known for its persistent elevation during chronic inflammatory states, including epilepsy [1, 26, 58]. IL-1β, in particular, plays a key role in driving neuroinflammation [60], and its reduction suggests that Boldo effectively mitigates one of the central mediators of the inflammatory response. Likewise, the normalization of TNF-α and MDA levels further supports Boldo’s capacity to counteract both inflammation and oxidative stress. These results underscore Boldo’s therapeutic potential in managing neuroinflammatory and oxidative components of epilepsy and suggest it may offer greater efficacy in these domains than standard VPA treatment. We recognize that cytokine measurements were performed in plasma rather than directly in brain tissue due to limited tissue availability. However, plasma cytokine levels have been widely validated as reliable indicators of systemic and central neuroinflammatory states in PTZ-kindled models [12, 54]. Inflammatory plasma cytokines are also elevated in patients with active epilepsy [30, 53, 94], indicating their potential as biomarkers for epilepsy. In this context our results support the idea that Boldo treatment reduces the inflammation associated to epilepsy. However, experiments directly assessing cytokines in brain tissue are recommended to complement these findings, but the current plasma data provide robust evidence of Boldo’s impact on neuroinflammation.

Collectively, our findings suggest that the therapeutic efficacy of VPA is significantly improved when combined with an HC blocker, such as Boldo. We have shown that Boldo leaves effectively blocked HCs in astrocytes, microglia, and neurons. This conclusion is further supported by the observation that an aqueous infusion of Boldo leaves inhibits and acutely blocks (within 5 min) both Cx43 and Panx1 HCs, whose activation is increased in hippocampal astrocytes, microglia, and neurons in the epileptic model demonstrated here. This enhanced HCs activity could exacerbate seizures by releasing glutamate and ATP, thereby increasing neuronal excitability. Intriguingly, this blocking capacity is observed despite the minimal water-soluble boldine content in the infusion [29, 81]. This suggests that Boldo's antiepileptic properties extend beyond the activity of boldine alone.

The antiepileptic effect of Boldo could also be explained by the combination of other active principles present in their leaves, such as gallic acid, or flavonoids as catechin, and quercetin, whose antiepileptic effect has been reported [48, 75, 96], and concentrations of these compounds, including boldine, were measured by HPLC in Boldo leaves. In experimental models, Moezi et al. [62] found that boldine at 50 mg/kg increased the latency to PTZ-induced myoclonic seizures, while 10 mg/kg was effective against seizures induced by electroshock [61]. However, according to our HPLC data (Table 1), boldine content in Boldo leaves ranges from 0.030–0.035%, meaning that 50 mg of Boldo leaf powder would contain only about 0.015–0.0175 mg of boldine—approximately 700 times less than the doses used in Moezi's studies. This large discrepancy suggests that other compounds in Boldo may contribute more significantly to the anticonvulsant effects, or that a synergistic interaction among constituents could be responsible. For instance, catechin, also present in small amounts (estimated at 0.01–0.02 mg per 50 mg of Boldo), has shown antiepileptic activity in animal models at doses of 100 mg/kg [2]. Boldo has been used for centuries in Chilean traditional medicine and is officially recognized by the Chilean Ministry of Health. It is readily available in retail formulations (e.g., capsules, tablets). Its traditional use for liver ailments has also garnered recent scientific interest. A study reported that 2.5 g of dry Boldo per day, administered cyclically, prolonged cecal transit time in asymptomatic volunteers without adverse effects [38]. Moreover, the European Scientific Cooperative on Phytotherapy (ESCOP) has not identified any known drug interactions associated with Boldo.

Our results suggest that Boldo’s inhibition of HCs may contribute to its antiepileptic effects. Although direct analysis of Cx43 protein expression in brain tissue was not performed due to limited tissue availability, our functional Etd + uptake assays provide strong evidence for Cx43 hemichannel involvement, as Etd + uptake has been shown to strongly correlate with Cx43 hemichannel activity [70, 71]. Importantly, Boldo selectively blocked HCs without affecting Cx43 gap junctional communication. However, the extract contains multiple bioactive compounds with known antioxidant, anti-inflammatory, and antiepileptic properties, including boldine, catechin, quercetin, and gallic acid, which likely act synergistically. This multifactorial action likely explains the robust efficacy of Boldo in reducing seizures and neuroinflammation. Future studies using specific HC inhibitors, such as Gap19, could help clarify the relative contribution of HC blockade versus other constituents.

The results highlight Boldo 's potential as a therapeutic agent for epilepsy, contingent on standardized dosage, administration, and treatment duration. Utilizing a conversion factor between mouse and human lifespans [22], our 5-day treatment in epileptic mice translates to approximately 200 days in humans. We advocate for future clinical investigations to evaluate Boldo as a supplementary therapy alongside standard ASMs. This recommendation is based on several factors. Firstly, we found that the combination of Boldo and VPA yielded the most effective reduction in astro- and microgliosis. Secondly, ASMs remain a cornerstone of epilepsy management, effectively controlling seizures in roughly 70% of patients by influencing neuronal excitation and inhibition. Therefore, integrating Boldo to block HCs in both glial and neuronal cells can address neuroinflammation, potentially leading to reduced seizure burden, decreased neuronal loss, and a mitigation of adverse effects associated with ASMs.

Finally, while no overt toxicity was observed in our study, including weight loss or abnormal behavior, a comprehensive pharmacokinetic and safety evaluation of Boldo remains necessary. Previous clinical and preclinical [4, 73] reports support the hepatic and renal safety of Boldo at much higher doses than those used here, but future studies should incorporate direct assessments of liver and kidney function to further validate its translational potential.

## Conclusions

This study demonstrates that Boldo exerts significant antiepileptic, anti-inflammatory, and antioxidant effects in a PTZ-induced mouse model of epilepsy. We show that PTZ kindling elevates HC activity in astrocytes, microglia, and neurons, contributing to increased neuroinflammation and seizure severity. Boldo treatment effectively reduced glial reactivity, reversed key proinflammatory and oxidative stress markers (IL-1β, IL-6, TNF-α, and MDA), and inhibited HCs. However, these effects likely result from the combined action of multiple bioactive compounds in the extract rather than HC inhibition alone, highlighting a multifactorial mechanism. These actions allowed Boldo to outperform the standard antiseizure medication valproate in several aspects.

Importantly, while valproate (VPA) effectively reduced seizure severity and systemic inflammation, it failed to attenuate microgliosis and, in some cases, exhibited proinflammatory effects, likely due to its direct activation of hemichannels (HCs). Notably, the combination of Boldo and VPA significantly reduced microgliosis, an effect not observed with VPA alone. In terms of astrogliosis, all treatment groups—Boldo alone, VPA alone, and their combination—produced significant reductions, with a more marked trend observed whenever Boldo was present. These findings support a multifactorial mechanism in which Boldo’s constituents, including but not limited to boldine, acting synergistically to reduce neuroinflammation, oxidative stress, and seizure severity.

Given Boldo 's established safety in traditional and clinical contexts, its ready availability, and minimal adverse effect profile, our findings strongly support further preclinical and clinical investigation of Boldo —particularly in combination with existing ASMs—as a novel strategy to enhance epilepsy management by addressing both seizures and the underlying neuroinflammatory environment.

## Supplementary Information

Additional file1 (DOCX 408 kb)

## Data Availability

The datasets during and/or analysed during the current study available from the corresponding author on reasonable request.

## Abbreviations

ACFS: Artificial cerebrospinal fluid
ASM: Antiseizure medications
Cx: Connexin
DAPI: 4′,6-Diamidino-2-phenylindole
DCFS: Divalent cation-free solution
DMEM: Dulbecco's Modified Eagle's Medium
Etd+: Ethidium bromide
FBS: Fetal bovine serum
GABA: γ-Aminobutyric acid
HC: Hemichannel
HeLa Cx43: HeLa cells stably transfected with connexin 43-EGFP
HeLa Panx1: HeLa cells stably transfected with pannexin 1-EGFP
MDA: Malonaldehyde
MS: Mechanical stretch
Panx: Pannexin
PBS: Phosphate buffered saline
PTZ: Pentylenetetrazol
ROI: Region of interest
TLE: Temporal lobe epilepsy
VPA: Valproate
