# Supplementary material for: Boldo leaves reduce seizures, neuroinflammation, and hemichannel activity in a murine model of chronic epilepsy
Source: Biol Res. 2025 Dec 7;58:72. doi: 10.1186/s40659-025-00647-w (PMC12683898; doi:10.1186/s40659-025-00647-w)
Supplement: Supplementary file 1 — Additional file1 (DOCX 408 kb) [file 40659_2025_647_MOESM1_ESM.docx]

**Boldo Leaves Reduce Seizures, Neuroinflammation, and Hemichannel Activity in a Murine Model of Chronic Epilepsy**

Claudia García-Rodríguez^a^_1_, Carolina Flores-Muñoz^a^, Paola Fernández^a^, Marcela Escobar^b^, Álvaro O. Ardiles^a,c^, Ana M. Cardenas^a^, and Juan C. Sáez^a^

^a^Instituto de Neurociencias, Centro Interdisciplinario de Neurociencias de Valparaíso, Universidad de Valparaíso, Valparaíso, Chile.

^b^Facultad de Farmacia, Universidad de Valparaíso, Valparaíso, Chile.

^c^Facultad de Medicina, Escuela de Medicina, Universidad de Valparaíso, Valparaíso, Chile

_1_Present address: Instituto de Biomedicina de Sevilla (IBiS), Hospital Universitario Virgen del Rocío/CSIC/Universidad de Sevilla, Seville, Spain.


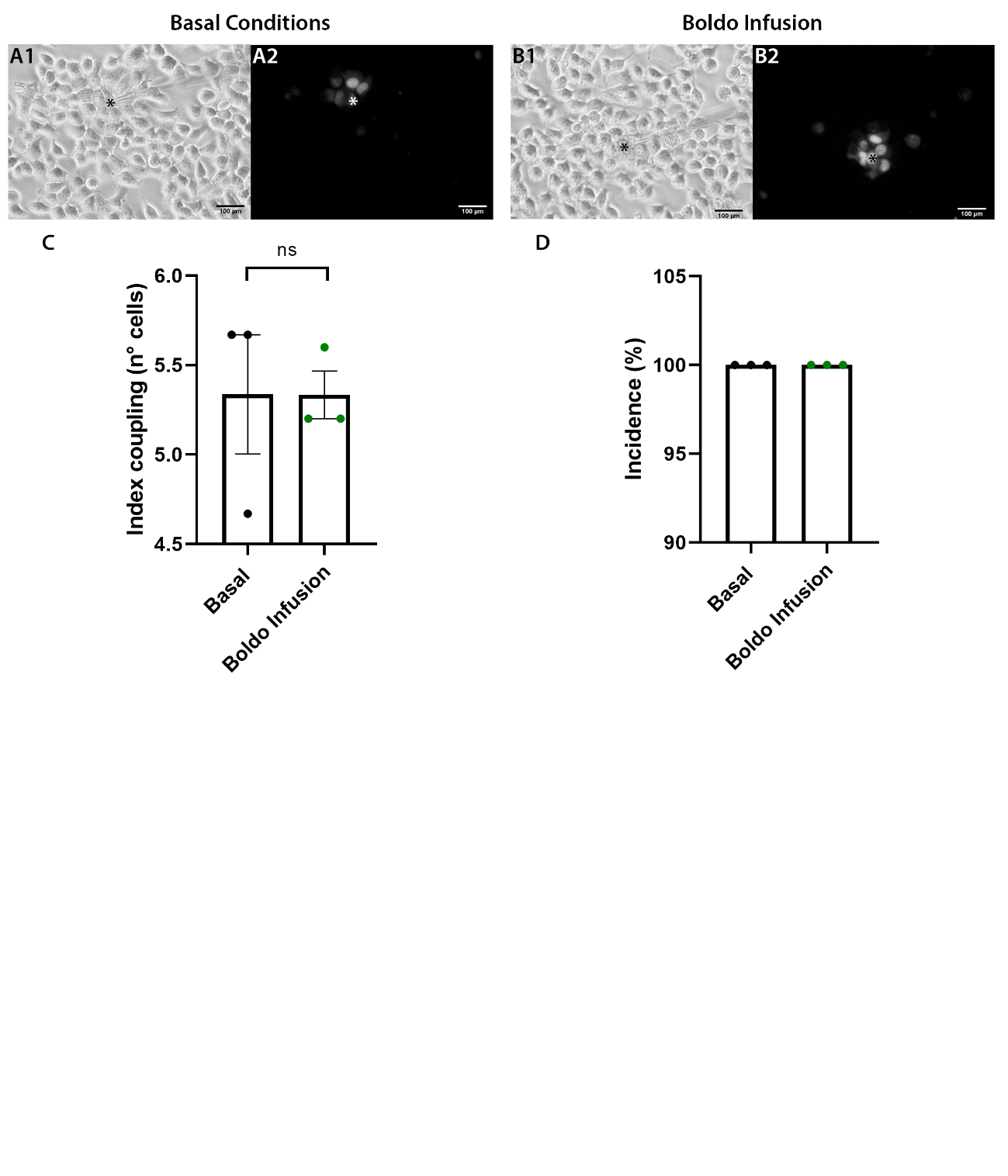


**Figure S1. Boldo infusion does not change the activity of intercellular channels mediated by gap junctions formed by connexin 43.** Sub-confluent cultures of HeLa Cx43 cells were used to perform dye-coupling experiments to measure the activity of GJCs. **A1.** HeLa Cx43 cells used. The seal is made with the cell marked with an asterisk. **A2.** Fluorescence of Etd+ after 5 min after micro ionizing the asterisk-labeled cell under basal conditions. **B1.** HeLa Cx43 cells used. The asterisk-labeled cell was microinjected. **B2.** Etd+ fluorescence after 5 min after microinjection of the asterisk-labeled cell in the presence of boldo infusion (Boldo leaves Suprema brand, 0.768 g/L). **C.** Coupling index under basal conditions and in the presence of boldo infusion (*n* = 3). Data correspond to mean ± SEM. A two-tailed unpaired Student's t-test was performed to compare between 2 conditions. ANOVA summary report: F (2, 2) = 6.250, *p*-value: 0.9930, *p*-value summary: ns, R^2^: 2.155e-005. **D.** Percentage of incidence in basal conditions and in the presence of boldo infusion (*n* = 3). No statistical analysis was made because the data were completely the same (100% incidence in both conditions).
